# Professional Shortcomings

**Published:** 1871-04

**Authors:** J. H. Foster

**Affiliations:** before the "American Academy of Dental Sciencs," at the Third Annual Meeting.


### ARTICLE II.

### *Professional Shortcomings.*

A portion of an Address delivered by J. H. Foster, M. D., before the "American Academy of Dental Science," at the Third Annual Meeting.

There are several points regarding professional shortcomings, upon which I shall be plain spoken.

*First* and foremost in the sins of omission, then, may be classed that of an unhealthy condition of the mucous membrane of the mouth,—arising from neglect in not properly removing, as often as necessary, the accumulation and deposit of tartar, affecting alike injuriously both gums and teeth,—and not instructing the patient how best to secure a comparative immunity from disease, by the right way of using brushes, wood-pointed sticks, floss silk, etc. It is no uncommon thing to see this general sanitary condition disregarded in a mouth where other operations of great merit have been performed. In an economical point of view, if no other, it is of great importance to the patient, If, then, this operation is neglected, because the fee is disproportionate to the time consumed, why not equalize it? For both patient and operator this ounce of prevention will be worth incomparably more than the pound of cure.

Next in importance is the neglect to institute stated critical examinations of the teeth, dependent in frequency upon their character, structure, and organization, in different individuals, particularly at an early age. Buccal surfaces of the Posterior Molares, not reached by the brush, and which once requiring to be filled, prove not only the most difficult operations to be performed, but the most uncertain, the same cause continuing to produce the same effect, unless the *preventive* course is pursued. How much better that it should be done before disease attacks them, by admonishing the patient, and imperatively stating the necessity for extreme attention to remote surfaces!

Another oversight is the neglect to discover disease until the enamel breaks in. This involves so much increased suffering to the patient, as well as difficulty to the operator, in the extra labor, time, and skill required, that more attention should be given to examinations; and if the operator considers it labor lost, let him charge a fee for his time. It would not, I think, be grudged by the patient, in view of immunity from present suffering, and the comforting assurance of security to his or her teeth for a still longer period.

It should also be considered a sacred duty on the part of the operator to instruct his patients how much their care may diminish the number of operations upon approximal surfaces, and, when the teeth have already been separated and filled, the necessity for keeping those surfaces clean and polished. The comparative durability of our operations depends much upon this, and earnest preaching may sometimes do as much good as practice, in ensuring a favorable result.

Carelessness in the mode of separating teeth, particularly the front incisors, is a point of grave consideration. Fillings cannot always be concealed, but often their edges, and sometimes a part of the fillings, are manifestly upon front surfaces, when they might have been under them.

No one would suppose that a man who had modelled and carved Artificial Teeth as a part of his education could ever lose sense of the importance of form and outline, and become unmindful or regardless of artistic skill in this respect, when operating upon the Natural Teeth. How common it is to see the latter mutilated and deformed, or so filed and cut away as to render subsequent operations extremely difficult and complicated, as if "some of Nature's journeymen had made men, and not made them well, they imitated Nature so abominably."

The great importance of rounding, blunting, and making smooth, sharp edges and points upon the grinding surfaces, formed by the wear and tear of antagonistic teeth, is too much disregarded,—these knife and saw edges forming weapons of offence alike destructive to the teeth and to the fillings in them. The much-abused File, gentlemen, is effective; and if called into requisition more frequently in such cases would prove a valuable auxiliary for the modification of this evil, in a short and summary way.

I would earnestly protest against a new method of separating teeth, for the purpose of filling cavities upon approximal surfaces, called instantaneous wedging, that is, driving wedges with a mallet above the affected surfaces, under the gum, between the necks of the teeth, with sufficient force to obtain the same room at once which it takes ten days or a fortnight to effect by the gradual, slow, and safe old method.

The shortest way of performing operations,— as in all other things, the *fastest time*,—seems to have become the aim and purpose, the great desideratum of the nineteenth century.

How comparatively few operators treat dead teeth, prepare and fill nerve cavities, in the most approved, thorough, and scientific manner, after the superior modern method! Who among us that ever practised the ancient barbarous method of destroying nerves is not thankful for the modern, humane, and painless way of performing the same operation?

I congratulate you, my brethren, that in our specialty, as in the practice of medicine, the necessity for the use of the lancet is almost entirely abolished. I have several of these little instruments laid aside, with the name of "Dixwell" upon them, purchased long ago at the sale of the effects of that old physician of your city. Who can tell how often they had been used in preceding years, in venesection, and how often since for opening abscesses consequent upon filling dead teeth after the old method, into which we were inducted by the most ancient and honorable of the profession?

Why, gentlemen, the old difficult way of withdrawing a charge from a gun, and the easy way with which its removal from a modern breech-loader is effected, is a just comparison. I have not opened an abscess, resulting from any fang operation of my own, for seven years, and might enter into a minute investigation of causes and effects, physiological and pathological, the consideration of which would occupy too much time.

Suffice it to say, that I consider this special success attributable to the *vis inertiœ*, the division and subdivision of the operation; never excising and removing the nerve until the sensibility is deadened by chemical agencies; never filling the nerve cavity the same day that the nerve has been excised; never filling the crown cavity the same day on which the fang is filled, and at no sitting continuing the operation after a feeling of soreness is induced by the irritation of working upon the tooth; but applying kreosote to the interior, as a counter-irritant, close the external cavity for the time, with "Hill's Stopping," and wait till another day. Teeth which have been long dead, and have absorbed into their structure offensive matter, require still more time for their purification. Some writers have attributed injurious effects to the continued use of kreasote. I have never seen such results, when it is applied in homœpathic quantities only, however oft repeated. Great importance should be attached to perfecting this operation at the apex of the fang; and when Pivot Teeth are engrafted upon fangs, the latter should undergo a similar expectant course of treatment, thorough and complete, that after evil consequences may not ensue, and need not even be apprehended.

I come now to a very important point,—a consideration of the treatment of *Six-year* or *First Permanent Molares.*

Is there one observant person among us, in practice for the last quarter of a century, who has failed to see the gradually increasing change in the organic structure of this particular tooth? Instead of being what it once was, a *permanent* tooth, has it not become, to all intents and purposes, deciduous? This I affirm to be the general rule. Of course there are exceptions, but they only prove the rule, and are continually decreasing in number. In all its characteristics, color, external and internal composition, and organic structure, it has become a *temporary* tooth; and in a majority of cases has less power to resist the action of those agencies which attack and destroy these organs than the temporary teeth have been supplied with. We daily see them crumbling away in the first year of their development, under influences which they were once as powerful to resist as any of the other permanent teeth, nay, more so than the Bicuspides.

Some have been inclined to dispute this position, and defend these poor teeth, which have no defensive armor themselves. Others deny that they are weak subjects, and insist that with proper treatment they may continue as long-lived as the rest. But "facts are stubborn things." The only question is, what, under the circumstances, is the specific? What is to be the mode of treatment? My own convictions, matured and strengthened with every year, have become settled and established modes of practice.

I trust, however, that no one will be influenced to adopt any system advocated and urged by another, except by evidence based upon the result of his own practical observation; for

> "He that complies against his will
> Is of his own opinion still."

Such converts are always clogs in the way of advancement.

I have no disposition to be considered "Sir Oracle," and desire only to awaken all to a consideration of matters having an important bearing upon professional responsibilities; to provoke agitation, excite discussion, and stimulate a desire for disputation even; for out of this come light and truth.

Concisely, then, as to the treatment of the Six-year or First Permanent Molares, they should be *extracted* in a large majority of cases.

Admitting this rule, how are we to know the exceptions? Is there any definite period, any stated time, when this operation should be performed? I answer, *beyond certain limits*, no! It will not do to act upon the principle, "If t'were well t'were done, when t'is done, t'were well t'were done quickly." We must gather wisdom by observation and experience. Pursue an expectant course of treatment, so as to give them trial. Prove them, and learn whether their ultimate retention will be for good or evil. At all events, the attempt should be made to guard against their loss until the "Second" or "Twelve-year Molares" shall have been developed; and if we find the latter so far back as to be predisposed to disease, from inability on the part of the patient to reach them effectually with the brush, or a crowded and irregular condition of the teeth generally, thereby inducing disease, upon the approximal surfaces, *then* their extraction is *imperative*. In how many cases do we fail to see this combination consummated?

Great consideration should be given to the theory advanced some years ago, by one of my honored instructors, Doctor Joshua Tucker, and afterwards endorsed by Dr. O. W. Holmes and others, that "the jaws of the Anglo-Saxon race have contracted, and that, whereas there was originally room for sixteen teeth in each jaw, there is now room but for fourteen."

The *Wisdom Tooth* proves much more reliable, if the First Molares have been extracted early; the Second Molares having moved forward to fill the vacancy, it takes the position of the latter, and having thereby additional room to develop, becomes a much more durable substitute.

Many persons still practice upon the supposition that the First Molares can and should be retained, under any and all circumstances, and insist upon pursuing the old theory of disregarding the preservation of the Wisdom Teeth. But if the front and lateral teeth are crowded and irregular, how is this *high pressure* to be reduced by persisting in this false theory and practice? I do not hesitate to affirm that any man, who ignorantly or wilfully shuts his eyes to the necessity of a *judicious thinning out*, in the mouths of young patients, may increase twofold the number of their operations in successive years, and incur the risk of losing both Six-year and Wisdom Teeth, besides increasing the chances of having to renew his fillings again and again, until finally, perhaps, in advanced life, his patients may find themselves in this condition, to use a scriptural quotation, "And behold seven thin ears, and blasted with the east wind." And he may then remorselessly cry out, "'Tis Cæsar's sword has made Rome's senate little, and thinned its ranks."

You will perceive, gentlemen, that I lay great stress upon this point. It is the *most important* one. More may be done in the way of prevention, by a judicious and wise course in this respect, than in any other.

When, in former years, it was deemed essential to advise the extraction of teeth in either jaw, in consequence of too crowded a condition, the response on the part of patients, or friends was "We will not consent to have sound teeth extracted." It seems to be an all wise dispensation of Providence that in view of the proportionate increase of disease at an early age, these Six-year Teeth have become what they are, so that we can imperatively say, under certain conditions, there is no use in trying to keep them, and the decrease of disease in the other teeth depends upon their extraction. But, alas! how many weak-minded, procrastinating, subservient men there are, ready to yield to solicitation or dictation, from mistaken motives of self-interest, or the promptings of a compassionate but injudicious humanity! "Fiat justitia, ruat cœlum."

In the way of *professional responsibility*, there should be no hesitancy, no doubt, no misgivings; and he who decidedly asserts and upholds his convictions will most surely receive the confidence of his patients, as well as maintain his own self-respect.

The popular cry, we know is, "Oh, we never extract permanent teeth; it is unnecessary,—*we* can save them! The *old fogies* advocate and practice such a course, but it is not at all in our way." What sympathizing parent is not most willing to be governed by this advice, to save a child the real and imaginary agony of extraction, bestowing at the same time a grateful regard upon *so considerate* a professional adviser? This delusion, however, must be short-lived, and woe to him who is thus influenced, and says, like Richard, "I have set my life upon a cast to stand the hazard of the die;" for ultimately he will surely die in professional *caste* and *reputation*.

Many there are, at the present day, who build superstructures upon such basis, at an immense cost, which are beautiful to behold, admirable specimens of artistic skill, and, if the substructure were only as perfect, might continue durable and serviceable; but, alas, too often there is no foundation, and the architect must be likened unto "A foolish man which built his house upon the sand; and the rain descended, and the floods came, and the winds blew, and beat upon that house; and it fell and great was the fall of it."

In concluding, a few remarks of a sanitary nature regarding ourselves may not be amiss. Most of us are overworked; and excess of work involves the need, particularly as we advance in life, of regular intervals of relaxation. Few of us are disposed to be governed by wisdom or common sense in this matter, until it is too late. It is a very poor economy, not to strengthen and utilize the physical power that God has given to man, to work out the term of his natural existence. None of us should be restrained from taking a long vacation from the fear of losing business. An earnest, conscientious devotion to the interests of our patients, will earn from them a grateful appreciation of our labors, and the pressing need of resting from them for a season. How many there are, alas, who rest from their labors eternally, whose lives and usefulness might have been extended and multiplied!

When the old woman told her doctor that "she feared her health was failing, because, on reading the newspapers, she did not enjoy the murders as she had done," she caught a glimpse of a great principle. When you find health and spirits are failing, natural cheerfulness and interest in your work decreasing,—that you can no longer "Smile and smile, and torture while you smile," be sure your experience is in similitude that of the old woman's, and you are beginning to have an insight into the eternal fitness of things which govern the laws of hygiene. *Then* it will be wise for you "to take thought and know yourself, and stay your fingers in good time."

I cannot close without saying a few words upon one other subject, which has an important bearing upon professional character and responsibility. Though not a *prohibitionist*, I am an advocate for temperance at all times and in all things. A sense of professional responsibility and self-responsibility and self-respect should impel every one rigidly and strictly to observe and practise *total abstinence* from everything that simulates or inebriates *during business hours.* There is the greatest need of caution in this direction. The continuation and the winding up of an honorable professional career may hang upon this thread, and it can be severed in a day, nay, in a single hour.

Unceasing vigilance, habitual prudence, and circumspection are indispensable if we would secure and maintain our principles; for even *appearances* are proofs sufficient to suspicious and malignant persons. However truly we may have kept the faith, however pure in motive and in acts our lives may be, "we ne'er can shape them so that evil-minded men will not their own construction put upon them." These *temperance* principles, feelingly and seriously urged, have been the inviolable rule of my own professional life, without exception; and I can point to their *strict observance* with the greater pride and satisfaction in the face of *false accusation* and *gross injustice.*

Finally, gentlemen, let us strive in every way to do all we can by precept and example to advance every good work, and to aid in encouraging institutions tending to this end. Let us believe in honesty of purpose; that no desires not bounded by the highest principle, no actions not guided by the light of conscience, can advance our true progress. There will always be individual cases of men governed by no principle or interest, except that kind of principle and interest which pertains to "the almighty dollar;" who make extortionate charges for their services whenever they can find subjects with more money than brains,—and the number of these is surprisingly large in every community. Such deluded persons are made to believe in the superior power, the magical achievements, the unequalled skill of their operator; and he whose real character is thus veiled by the illusion enjoys a short-lived prosperity, soon to be wafted away by the cold finger of truth and reality.

'Tis a trite remark, but one that forces itself upon us every day, that "gross deceit raises itself to high places, and sits in purple and fine linen, while Honesty is left to beg her bread, and plain Truth stands shivering in a blanket." Nevertheless the good and true man will not relax his exertions, from a despondent contemplation of the false success that stalks triumphantly in the walks of life. Nor will the pure-minded suffer his faith in human progress to decay and perish, because he cannot see that the world moves as his sympathy and hope would have it. "There is a silver lining to every cloud," and there is no well so deep but that the stars can be seen from its depths shining at midnight.

Let us, then, enlarge our confidence in the power to exercise a redeeming and reformatory influence over our fellow men; have more charity for their shortcomings, more patience with their infirmities, more faith, in the years to come, in the gradual and ultimate triumph of goodness, virtue, and truth, over corruption, vice and fraud.

These sad anomalies that we daily witness in civilized life, these painful incongruities that disturb our sense of right and wrong, may, after all, be appropriate discords in the grand harmony of universal music, arranged by the Divine master.

> "There's a divinity that shapes our ends,
> Rough-hew them how we will."

Let us, then, strive to be true and fearless, and to mix gentleness and love with fearlessness. Amid all mutations, and fluctuations of old schools and new, it will be the maximum measure of attainment that secures the vantage ground, and the honest, truthful, conscientious discharge of practical duty only that will ensure a memory and a name worthy to be enrolled on the pages of Time.

May this thought serve to awaken in all the desire to attain similar excellence, a like meed of praise, and the gratitude which blesses and sanctifies those who have exemplified in their lives a title to the appellation of a just man and a public benefactor.

